# Genome-wide linkage analysis using cross-sectional and longitudinal traits for body mass index in a subsample of the Framingham Heart Study

**DOI:** 10.1186/1471-2156-4-S1-S35

**Published:** 2003-12-31

**Authors:** Xiaohui Li, Dai Wang, Kai Yang, Xiuqing Guo, Ying-chao Lin, Carlos G Samayoa, Huiying Yang

**Affiliations:** 1 Division of Medical Genetics, Medical Genetics Birth Defects Center, Cedars-Sinai Research Institute, Cedars-Sinai Medical Center, Los Angeles California, USA; 2 University of California, Los Angeles, California, USA

## Abstract

To evaluate linkage evidence for body mass index (BMI) using both cross-sectional and longitudinal data, we performed genome-wide multipoint linkage analyses on subjects who had complete data at four selected time points (initial, 8^th^, 12^th^, and 16^th ^year following the initial visit) from the Framingham Heart Study. The cross-sectional measures included BMI at each of the four selected time points and the longitudinal measure was the within-subject mean of BMI at the above four time points.

Using the variance components method, we consistently observed the maximum LOD score out of the genome scan using BMI at each time point and the mean of BMI between 049xd2 and GATA71H05 on chromosome 16. The highest LOD score (3.0) was at time point 1, while the lowest (1.9) was at time point 4. We also observed other suggestive linkages on chromosome 6, 10, and 18 at time point 1 only.

The longitudinal measure we studied (mean of BMI) did not provide greater power to identify a positive linkage than some of the cross-sectional measures (e.g., time point 1). The changing of linkage evidence over time provided some insights on the variation of genetic effect on BMI with aging. There may be a QTL on chromosome 16 that contributes to BMI and this locus, and maybe others, is more likely to affect BMI during early adulthood.

## Background

Obesity, which is influenced by both genetic and environmental factors, is an independent risk factor for coronary heart disease (CHD). However, the reported quantitative trait loci (QTLs) for body mass index (BMI), which is one of the obesity measures, are not consistent. The discrepancies among various studies represent the difficulties in identifying susceptibility genes for common complex traits and may be caused by any of the factors affecting the ability to detect linkage. These factors include, but are not limited to, sample sizes, ascertainment methods, long-term or short-term environmental influences, and genetic heterogeneity. In addition, variable age-dependent expression of a trait can also affect our ability to identify corresponding susceptibility genes. A longitudinal study design will provide a unique opportunity to evaluate such age related effects.

However, due to their relative feasibility, cross-sectional designs using phenotype data collected at one time point are used in most linkage studies. As compared with the cross-sectional design, longitudinal data may be able to answer certain questions that cross-sectional data can not. For example, genes that determine change of a trait over time (e.g., slope of BMI) can only be identified through a longitudinal design. Furthermore, serial observations over time of the same trait may allow more accurate partitioning of genetic and environmental components than would a single observation [1]. Thus, longitudinal data may provide more insight to dissect genetics of a complex trait.

The Framingham Heart Study is a very successful longitudinal study of cardiovascular diseases that was started in 1948. This study has obtained vast longitudinal cardiovascular-related phenotypic measures in two generations of participants. A 10-cM density genome-wide scan in subjects from 330 families was finished in the late 1990s. This provided a unique opportunity to identify QTLs for CHD-related traits using longitudinal phenotypes.

Our goals in this study were 1) to identify the QTLs for BMI using multipoint linkage analyses and 2) to assess the consistency of QTLs identified over time using the Framingham Heart Study data.

## Methods

### Subjects

The original cohort in the Framingham Heart Study was recruited in 1948: 5209 subjects were followed up every 2 years, with a total of 21 visits. In 1971, the study enrolled an additional 5124 adult children of the original participants and spouses of these children as the offspring cohort. This offspring cohort was followed up at 8^th^, 12^th^, 16^th^, and 20^th ^years after their initial visit, for a total of five visits. A total of 330 extended pedigrees consisting of 4692 subjects were selected for the genome scan in the Framingham Heart Study. Longitudinal phenotype measurements were available for 2885 subjects; 1712 had genotype data.

To make the linkage analysis results from different time points comparable, we analyzed only those subjects with complete phenotypic data at all four selected visits. In addition, only families with at least two individuals (non parent-offspring relationship) who met such criteria were included in the analysis.

### Phenotype

BMI [weight (kg)/ height (m)^2^], was selected as the quantitative trait in our analysis because of its completeness and uniform measurement method over time. Data collected in year 1971, 1979, 1983, and 1987 from both the original cohort (visit 11, 15, 17, 19) and the offspring cohort (visit 1, 2, 3, 4) were denoted as BMI1 – BMI4, respectively. The within-subject mean of the four visits over 16 years, denoted as MEAN, was also generated for analysis.

### Linkage analysis

Linkage analyses were conducted using the variance components analysis method as implemented in the SOLAR program [2]. This method evaluates linkage by comparing a variance component model that permits a particular locus to account for some of the additive genetic variance (along with a residual polygenic component) to a purely polygenic model by using likelihood ratio tests. The estimates of shared identity by descent (IBD) for individual markers were provided by GAW13. Multipoint estimates of IBD were obtained as weighted average of IBD for individual markers by using the SOLAR program.

Multipoint linkage analysis for quantitative traits was performed on BMI1 – BMI4 and the MEAN. The covariates evaluated in the linkage model were age, gender, and cohort. A covariate was retained in the linkage analysis model only if it reached significance at a level of 0.05.

## Results

Table 1 shows the general characteristics of the analyzed sample at time point 1 and time point 4 (16 years later). In the selected sample, there were 1502 subjects from 291 pedigrees, including 808 females and 694 males. The actual number of subjects used in the linkage analysis may be reduced due to missing IBD information. The mean age at time point 1 was approximately 40 years old. The mean values of BMI were 27.3 for male and 25.0 for female, while they increased to 28.2 and 26.7 at time point 4, respectively.

**Figure 1:**
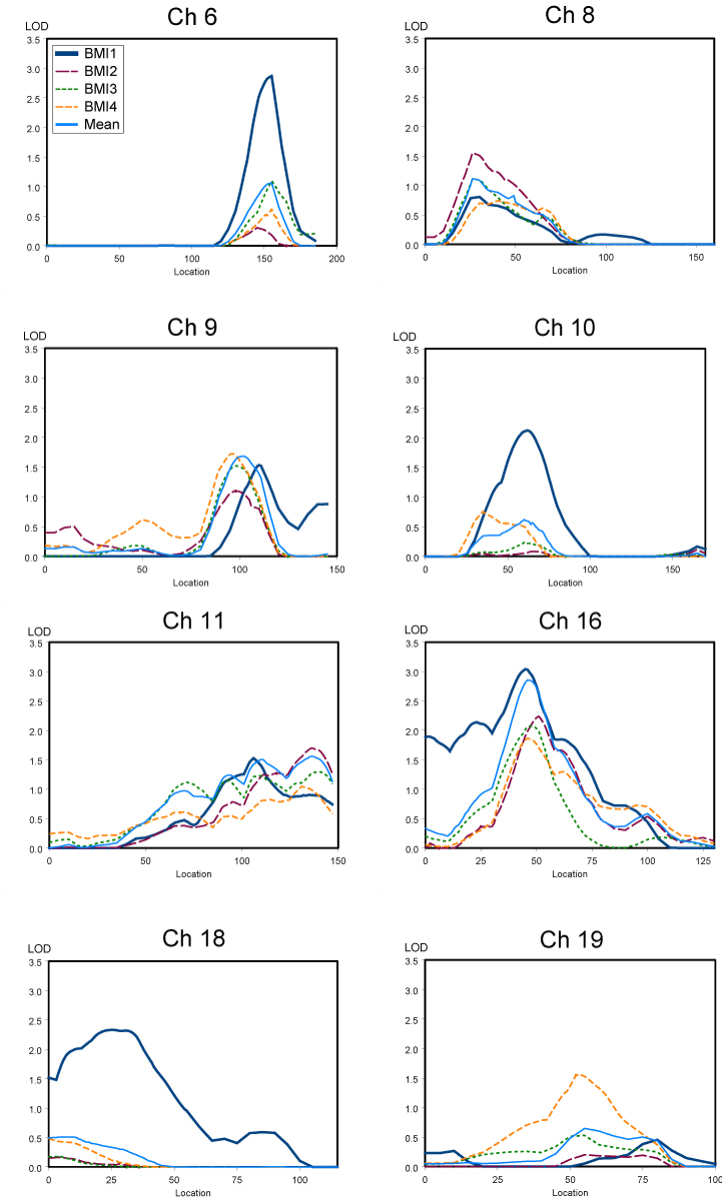
Selected chromosomes from genome scan results for BMI

Figure 1 shows the selected chromosomes from genome scan linkage analysis for BMI1 – BMI4 as well as the MEAN. These chromosomes have maximal LOD score greater than 1.3 observed on at least one of BMI1 – BMI4 or on the MEAN. In general, the peaks identified from BMI1 – BMI4 and the MEAN are localized within the same regions, although the magnitude of LOD score may vary. The maximum LOD score out of the genome scan at each time point was consistently observed between 049xd2 and GATA71H05 on chromosome 16 (Table 2), with the highest LOD score at time point 1 (3.0) and the lowest at time point 4 (1.9). Three other suggestive linkages (LOD score ≥ 2.0) were also observed at time point 1, while neither the data from other time points nor the MEAN reached suggestive linkage level. Suggestive linkages were located at chromosome 6 near GATA165G02 (LOD = 2.9 at 155 cM), chromosome 10 near ATA5A04 (LOD = 2.1 at 62 cM), and chromosome 18 near ACT1A01 (LOD = 2.3 at 25 cM).

## Discussion

We performed genome scan linkage analyses for BMI measured at four different time points (over 16 years) and the mean of BMI over the four time points in a select sample from the Framingham Heart Study. The strongest evidence of linkage was observed on the short arm of chromosome 16 near centromere (16p11.2-12) at time point 1 (LOD = 3.0). Although this LOD score does not quite reach the genome-wide significant linkage level (3.3), we observed the same maximum peak with all other time points and the MEAN, with the lowest LOD score at time point 4 (LOD = 1.9). Using the same Framingham Heart Study data, Atwood and colleagues detected two linkage regions on chromosome 6 and 11 [3]. The maximum LOD score was 4.6 in the chromosome 6q23-25 region at time point 1. We observed a suggestive linkage with a LOD score of 2.9 at time point 1 in the same region. The obvious difference between our analyses and that of Atwood et al. is in sample selection. Atwood et al. used all available data at time point 1, while we selected a subset from the total sample, which has complete data at all four time points. By comparing the age (mean: 40.4 vs. 40.8) and BMI (mean: 25.5 vs. 26.0) distributions between Atwood et al.'s and our samples, there seems to be no obvious difference between the two samples. Therefore, the difference in the magnitude of LOD scores at 6q23-25 could be due to sample size difference. However, it is not clear why evidence for linkage at 16p11.2-12 was not identified in Atwood et al.'s analysis.

It is intriguing that the highest LOD score at 16p11.2-12 was observed at time point 1, while the lowest was at time point 4, and that three other suggestive linkages were all observed at time point 1. Since all subjects have data on all time points, the only difference between each time point is age and BMI values. Time point 1 captured a younger stage of the same population as compared with later time points. The observations that the maximum LODs decreased over time, and that other suggestive linkages were observed only at time point 1 suggest that these QTLs may have greater influence on BMI during the early years of life. Such variations of genetic influence with age may be further supported by a few available heritability studies of BMI. In a study of the National Heart, Lung, and Blood Institute male veteran twins, Fabsitz et al. estimated a heritability of 82% at age 20 and observed a somewhat reduced heritability after age 48 (72–78%) [4]. They also suggested that different sets of genes may be active at different age periods. Similarly, in the longitudinal Quebec Family Study [5], maximal heritability (2 × r_sibling_) at time 1 was higher (44%) than 12 years later (36%). These authors also suggested that some familial factors affect BMI consistently overtime and some additional familial factors affect BMI at different times. If such age-related heritability variations are true, and they are determined by different genes, it is conceivable that certain genes can only be identified during certain periods of life, for example suggestive linkage at 6q23-25 was only observed at time point 1. The consistent location of the maximum LOD score for BMI over time on 16p11.2-12 in our study indicates that this putative QTL has a relatively lasting effect on BMI, while such effect may be veiled by environmental factors over time. The chromosome 16 locus may be a true susceptibility locus for BMI, but this is the first report of this locus so an independent confirmation would increase its validity.

Using longitudinal observations of the same trait, BMI, we derived MEAN as a longitudinal phenotype that represents an overall status of BMI across 16 years. However, MEAN did not improve LOD score at either the previously reported 6q23-25 location or at our best peak on chromosome 16. One explanation could be that the effects of these QTLs were reduced at later years of life so that the composite measure, MEAN, from all age periods averaged out the effect of these QTLs. Although in this study, this longitudinal design did not provide more power to identify QTLs for BMI than a single cross-sectional data at time point 1, without longitudinal data, we would not be able to observe variations of genetic effect overtime. With such knowledge, in order to increase power to identify QTLs for BMI, we may design future linkage studies by ascertaining families with young adults. However, keep in mind that the effects of different QTLs and environmental factors may be different during a lifetime. When we restrict sample selection, we may gain power to identify certain genes but may not be able to identify others.

In summary, BMI varies with age, and different genes may determine variations in the population at different age periods. For example, genes that influence childhood obesity may be different from genes that influence adulthood obesity. Nevertheless, when we analyzed cross-sectional BMI data at different time points and the mean of all time points, we observed a consistent linkage with BMI at 16p11.2-12 across all time points, and three other suggestive linkages, including the previously reported 6q23-25. In addition, we observed variation in LOD score over time with the highest at time point 1 and the lowest at time point 4 (16 years later). These results indicate that there may be a QTL on chromosome 16 that contributes to BMI and this locus, and maybe others, is more likely to affect BMI during early adulthood.

## Figures and Tables

**Table 1:** Characteristics of the analyzed sample at time point 1 and time point 4

	Range		Mean ± SD		
		
	Min	Max	Male (*N *= 694)	Female (*N *= 808)	Total (*N *= 1502)
Time point 1					
Age	19	75	39.7 ± 13.0	41.8 ± 13.6	40.8 ± 13.4
BMI	14.0	47.9	27.3 ± 3.5	25.0 ± 4.3	26.0 ± 4.1
Time point 4					
Age	34	93	55.7 ± 13.2	57.8 ± 13.8	56.8 ± 13.6
BMI	14.1	52.7	28.2 ± 3.8	26.7 ± 5.3	27.4 ± 4.7

**Table 2:** The maximum LOD scores and additional suggestive linkage at each time point and the MEAN for BMI

Time	Maximum LODs		Additional suggestive linkage					
	
Point	Chromosome 16		Chromosome 6		Chromosome 10		Chromosome 18	
		
	Position	LOD	Position	LOD	Position	LOD	Position	LOD
1	45	3.0	155	2.9	62	2.1	25	2.3
2	51	2.2	145	0.3	65	0.1	5	0.2
3	48	2.1	155	1.1	60	0.2	5	0.2
4	46	1.9	155	0.6	35	0.8	0	0.5
MEAN	46	2.9	155	1.1	60	0.6	5	0.5
